# First Glycoside Hydrolase Family 2 Enzymes from *Thermus antranikianii* and *Thermus brockianus* with β-Glucosidase Activity

**DOI:** 10.3389/fbioe.2015.00076

**Published:** 2015-06-03

**Authors:** Carola Schröder, Saskia Blank, Garabed Antranikian

**Affiliations:** ^1^Institute of Technical Microbiology, Hamburg University of Technology, Hamburg, Germany

**Keywords:** β-glucosidase, glycoside hydrolase, thermostable, *Thermus antranikianii*, *Thermus brockianus*, cellobiose, truncated domains

## Abstract

Two glycoside hydrolase encoding genes (*tagh2* and *tbgh2*) were identified from different *Thermus* species using functional screening. Based on amino acid similarities, the enzymes were predicted to belong to glycoside hydrolase (GH) family 2. Surprisingly, both enzymes (*Ta*GH2 and *Tb*GH2) showed twofold higher activities for the hydrolysis of nitrophenol-linked β-D-glucopyranoside than of -galactopyranoside. Specific activities of 3,966 U/mg for *Ta*GH2 and 660 U/mg for *Tb*GH2 were observed. In accordance, *K*_m_ values for both enzymes were significantly lower when β-D-glucopyranoside was used as substrate. Furthermore, *Ta*GH2 was able to hydrolyze cellobiose. *Ta*GH2 and *Tb*GH2 exhibited highest activity at 95 and 90°C at pH 6.5. Both enzymes were extremely thermostable and showed thermal activation up to 250% relative activity at temperatures of 50 and 60°C. Especially, *Ta*GH2 displayed high tolerance toward numerous metal ions (Cu^2+^, Co^2+^, Zn^2+^), which are known as glycoside hydrolase inhibitors. In this study, the first thermoactive GH family 2 enzymes with β-glucosidase activity have been identified and characterized. The hydrolysis of cellobiose is a unique property of *Ta*GH2 when compared to other enzymes of GH family 2. Our work contributes to a broader knowledge of substrate specificities in GH family 2.

## Introduction

Glycoside hydrolases (GHs) (EC 3.2.1.X) hydrolyze glycosidic bonds between carbohydrates or between carbohydrate and non-carbohydrate components, for instance alcohols or phenols. To date, enzyme classification is based on amino acid similarities rather than substrate specificity (Henrissat, 1991). GHs are assigned to different groups based on conserved domains, and the families are numbered from 1 to 131 that are listed in the Carbohydrate-Active Enzymes Database^1^ (Lombard et al., 2014). Evolutionary or structurally unrelated β-glucosidases (EC 3.2.1.21) are found in six GH families (1, 3, 5, 9, 30, and 116). The majority of identified β-glucosidases is grouped in GH family 1 (Cairns and Esen, 2010). β-Galactosidase activity can be found in different GH families (1, 2, 3, 16, 20, 35, 42, 43, 50, and 59). In contrast to GH family 1, no β-glucosidase is found in GH family 2. So far, glycoside hydrolases classified as GH2 were described to function as β-galactosidase (EC 3.2.1.23), β-mannosidase (EC 3.2.1.25), β-glucuronidase (EC 3.2.1.31), endo-β-mannosidase (EC 3.2.1.152), or as exo-β-glucosaminidase (EC 3.2.1.165). These enzymes hydrolyze their substrates through a retaining acid/base mechanism with two glutamic acid/glutamate residues involved. One residue acts as acid and base catalyst, whereas the other residue serves as nucleophile (Davies and Henrissat, 1995).

Glycoside hydrolases find a wide range of industrial applications. For instance, β-glucosidases are applied in biorefineries to reduce cellobiose-mediated product inhibition of endoglucanases. Thus, the addition of β-glucosidases leads to increased glucose concentrations during the enzymatic hydrolysis of cellulose, thereby increasing ethanol yields (Viikari et al., 2007). Furthermore, β-glucosidases and β-galactosidases are applied in the food industry to improve the aroma of juices and wine (Bhatia et al., 2002). Many industrial processes run at harsh conditions, e. g., elevated temperatures and extremes of pH. Hence, the demand for novel enzymes which function at high temperatures or acidic or alkaline pH values is growing (Antranikian and Egorova, 2007).

Bacteria belonging to the genus *Thermus* grow at temperatures between 53 and 86°C, and at pH values between 6.0 and 10.5 (Dworkin et al., 2006). Since the discovery of the type strain of this genus, *Thermus aquaticus* in 1969, numerous species have been isolated from hot environments (Brock and Freeze, 1969; Oshima and Imahori, 1974). Especially, the DNA polymerase from *T. aquaticus* (*Taq* DNA polymerase) became one of the key enzymes in molecular biology (Chien et al., 1976). Furthermore, thermostable DNA-processing enzymes such as ligases, helicases, or endonucleases have been identified (Pantazaki et al., 2002). Although *Thermus* spp. produce different hydrolytic enzymes like proteases or lipases, only few glycoside hydrolases have been characterized so far (Dion et al., 1999; Fridjonsson et al., 1999; Pantazaki et al., 2002; Kim et al., 2006; Nam et al., 2010; Blank et al., 2014).

In the current report, two genes coding for GHs (*Ta*GH2 and *Tb*GH2) were identified from *Thermus antranikianii* and *Thermus brockianus*. Conserved domains of GH2 were detected with incomplete motifs. The recombinant proteins showed highest activity toward 4-NP-β-d-glucopyranoside. The activity of *Ta*GH2 toward cellobiose makes this enzyme unique when compared to the GH family 2.

## Materials and Methods

### Bacterial strains and plasmids

*Thermus antranikianii* and *Thermus brockianus* were used as potential sources for the detection of β-glucosidase-encoding genes. Both strains were obtained from the collection of bacterial strains of the Institute of Technical Microbiology, Hamburg-Harburg. The strains *Escherichia coli* XL1-Blue MRF’ [Δ(*mcr*A)*183* Δ(*mcr*CB*-hsd*SMR*-mrr*)*173 end*A1 *sup*E44 *thi-1 rec*A1 *gyr*A96 *rel*A1 *lac* [F’ *pro*AB *lac*IqZΔM15 Tn10 (Tet^R^)]] and *E. coli* XLOLR (Δ(*mcr*A)*183* Δ(*mcr*CB*-hsd*SMR*-mrr*)*173 end*A1 *thi-1 rec*A1 *gyr*A96 *rel*A1*lac* [F’*pro*AB *lac*IqZΔM15 Tn10 (Tet^R^)] Su-(non-suppressing) λr) were obtained from the “ZAP Express Predigested Vector Kit” (Agilent Technologies, Waldbronn, Germany), and were utilized to construct and screen phagemid libraries using the vector pBK-CMV.

*Escherichia coli* Nova Blue Single^TM^ (*end*A1 *hsd*R17(r^−^_K12_ m^+^_K12_) *sup*E44 *thi-1 rec*A1 *gyr*A96 *rel*A1 *lac* F’[*pro*A^+^ B^+^*lac*l^q^ ZΔM15:Tn10 (Tc^R^)]) (Novagen/Merck, Darmstadt, Germany) and *E. coli* BL21 Star^TM^(DE3) [F^−^ *omp*T *hsd*SB (rB-mB-) *gal dcm rne*131 (DE3)] (Invitrogen, Karlsruhe, Germany) were used as hosts for cloned PCR products and expression using the plasmids pJet1.2/blunt (Fermentas, St.Leon-Rot, Germany) and pQE-80L (Qiagen, Hilden, Germany).

### Media and culture conditions

The *Thermus* strains were grown at 70°C and 200 rpm for 16 h in *Thermus* 162 medium (DSMZ medium 878). *E. coli* strains were grown in LB medium at 30–37°C and 100–180 rpm for 16 h (Sambrook et al., 2001). LB medium was supplemented with selected antibiotics (ampicillin: 100 μg/mL, kanamycin: 50 μg/mL) when cultivating bacteria harboring appropriate plasmids. For gene expression, isopropyl-β-d-thiogalactopyranoside (IPTG) in concentrations of 0.5–1.0 mM were added.

### Isolation of DNA, construction and screening of gene libraries

Genomic DNA from *Thermus* sp. was isolated according to the “Genomic DNA Handbook” (Qiagen, Hilden, Germany), and further purified by phenol/chloroform/isoamyl alcohol-extraction (25/24/1) and ethanol precipitation. Plasmid DNA was isolated using the “GeneJET^TM^ Plasmid Miniprep Kit” (Fermentas, St. Leon-Rot, Germany).

Isolated genomic DNA from *Thermus* strains was partially digested with *Bam*HI to generate fragments of 5–10 kb. DNA fragments of the desired size were separated by agarose gel electrophoresis. To prepare an agarose gel of 1%, 1 g agarose was dissolved in 100 mL TAE buffer (40 mM Tris, 1 mM EDTA, 40 mM acetic acid, pH 8.5). The DNA was extracted from the gel by using the “GeneJet Gel Extraction Kit” (Fermentas, St. Leon-Rot, Germany). The ligation into the “ZAP Express Vector,” as well as further steps for gene library construction were carried out according to the manufacturer’s instructions.

To detect β-glucosidase-encoding genes, the gene libraries were plated on LB agar supplemented with 50 μg/mL kanamycin, and after growth over night the colonies were replicated on plates containing additionally 1 mM IPTG. The clones were overlayed with buffer (25 mM sodium acetate, 2.5 mM CaCl_2_ × 2 H_2_O, 170 mM NaCl, 1% agarose, pH 6.5) containing 2.5 mM esculin and 0.4 mM ammonium-iron(III)-citrate. After incubation at 70°C for 1–16 h, β-glucosidase positive clones were observed by the formation of a brown halo around the colonies*. E. coli* XLOLR pBK-CMV clones displaying β-glucosidase activity were conserved as cryostocks containing 25% glycerol.

### Sequencing and sequence analysis

Plasmids were isolated (“GeneJET^TM^ Plasmid Miniprep Kit”, Fermentas, St. Leon-Rot, Germany) from selected clones, which conferred β-glucosidase activity. To determine the DNA-sequence of integrated DNA-fragments, the plasmids were sent to Eurofins MWG Operon (Ebersberg, Germany) for sequencing.

Putative open reading frames (ORF) were detected using the program “FramePlot 4.0beta.”^2^ The DNA- and amino acid-sequences were compared to the database of the “National Centre for Biotechnology Information” (NCBI^3^) using the “Basic Local Alignment Search Tool” (BLAST) (Altschul et al., 1997). Conserved domains were predicted using “InterProScan” (EMBL-EBI^4^) and the Conserved Domain Database (Marchler-Bauer et al., 2013). Glycoside hydrolase family 2 signatures were retrieved from http://prosite.expasy.org/PDOC00531. For multiple sequence alignments, ClustalW2 was employed (EMBL-EBI^5^, 2013). Hypothetical models of protein structures, based on sequence homologies, were prepared with the program “SWISS-MODEL” (Gasteiger et al., 2003).

### Cloning of genes

To amplify the β-glucosidase-encoding genes, specific primers were obtained from Eurofins MWG Operon (Ebersberg). For the amplification of the gene *tagh2*, the primers *tagh2*_f_*Pae*I (5′-*GCATGC*AGGTGGGAAAGAGCTTGGTTTTTG-3′) and *tagh2*_r_*Sal*I*Hind*III (5′-*AAGCTTGTCGAC*TCACCAGGCCACCCCCAGGG-3′) were used, while *tbgh*_f (*GGATCC*AGGCTAAAAAGCGCCCTTTTC) and *tbgh2*_r (*GTCGAC*CTACCAAGCCTCTCCAGG) were employed to amplify the gene *tbgh2*.

The PCRs were carried out in a thermocycler according to the manufacturer’s instructions. A mixture of 20 μL contained 0.4 U “Phusion High-Fidelity DNA-Polymerase” (Fermentas, St. Leon-Rot, Germany), 0.2 mM dATP, dCTP, dGTP, dTTP, 0.5 μM of the forward and reverse primer, 10–300 ng template, reaction buffer, and distilled autoclaved water. The obtained PCR products were cloned into the pJet1.2/blunt vector (Fermentas, St. Leon-Rot, Germany), which was thereafter used to transform competent *E. coli* Nova Blue Single^TM^ cells. Positive transformants were identified by colony PCR. Isolated pJet1.2/blunt plasmids were double digested with *Pae*I/*Sal*I or *Bam*HI/*Sal*I to excise the β-glucosidase-encoding genes. The purified genes were ligated into the *Pae*I/*Sal*I and *Bam*HI/*Sal*I digested expression vector pQE-80L, which was used to transform competent *E. coli* BL21 Star^TM^(DE3) cells. Positive transformants were identified by colony PCR and conserved as cryostocks containing 25% glycerol.

### Production and purification of the enzymes

*E. coli* BL21 Star^TM^(DE3) harboring the recombinant pQE-80L plasmids were cultivated in 5 mL LB medium containing 100 μg/mL ampicillin at 37°C and 160 rpm for 16 h. The preculture was subcultivated in 500 mL LB medium containing 100 μg/mL ampicillin until the culture reached an optical density of A_600 nm_ = 0.5–0.7. To induce protein production, 0.5 M IPTG was added. The cultivation was continued for 6 h, afterwards the cells were harvested by centrifugation at 13,000 rpm for 20 min at 4°C. The cell pellet was resuspended in lysis buffer (50 mM NaH_2_PO_4_, 300 mM NaCl, 10 mM imidazole, pH 8) in the ratio 5 mL buffer/1 g pellet. Cells were disrupted by French press (French^R^ Pressure Cell Press, SLM-Aminco, MD, USA). Crude extracts with protein contents of 1.5 mg/mL (*Ta*GH2) and 1.07 mg/mL (*Tb*GH2) were obtained by centrifugation at 13,000 rpm at 4°C for 20 min.

For heat precipitation, the crude extract was incubated at 70°C for 15 min, and subsequently centrifuged at 13,000 rpm at 4°C for 20 min.

As a second purification step, a Ni^2+^-nitrilic acid (Ni-NTA) affinity chromatography using a 1.5-mL Ni-NTA superflow column (Qiagen, Hilden, Germany) was conducted. About 2–5 mL of the crude extract was incubated with 1 mL of Ni-NTA-agarose (Qiagen, Hilden, Germany) at 4°C and 200 rpm for 1 h. Two washing steps were performed (50 mM NaH_2_PO_4_, 300 mM NaCl, 25 mM imidazole, pH 7.0). To elute the protein, six fractions of 500 μL elution buffer (50 mM NaH_2_PO_4_, 300 mM NaCl, 250 mM imidazole, pH 7.0) were added to the column. Fractions containing the protein were pooled.

In the case of *Tb*GH2, a gel filtration using the “ÄKTA^TM^ Fast Protein Liquid Chromatography” (FPLC)-system (GE Healthcare, München, Germany) was carried out. One milliliter of the protein solution was load on a “HiLoad 16/60 Superdex 200 prep grade”-column (GE Healthcare, München, Germany) with a flow rate of 1 mL/min. The protein was eluted with a 1.5-fold column volume of 50 mM sodium phosphate buffer, pH 7.2, which contained 150 mM NaCl. Enzyme fractions were pooled.

The purified proteins were dialyzed against 20 mM citrate buffer (*Ta*GH2) or 20 mM maleate buffer (*Tb*GH2) and stored at 4°C.

The purity of the recombinant β-glucosidases was analyzed by sodium dodecyl sulfate polyacrylamide gel electrophoresis (SDS-PAGE, 12%) (Laemmli, 1970).

Protein concentrations were determined using the method described by Bradford (1976).

### Determination of enzyme activity

Enzyme activity was determined by the released amounts of 2-nitrophenol (2-Np) and 4-nitrophenol (4-Np) from several nitrophenol-linked substrates (4-Np-β-d-glucopyranoside, 4-Np-α-d-glucopyranoside, 4-Np-β-d-glucuronid, 4-Np-β-d-galactopyranoside, 2-Np-β-d-galactopyranoside, 4-Np-α-d- galactopyranoside, 4-Np-β-d-xylopyranoside, 4-Np-β-d-manno pyranoside, 4-Np-β-d-cellobioside).

The reaction mixture of 1 mL contained 2 mM Np-substrate and 20 mM citrate buffer pH 6.5 (*Ta*GH2), or 20 mM maleate buffer pH 6.5 (*Tb*GH2). After preincubation of the reaction mixture for 5 min at 95°C (*Ta*GH2) or 90°C (*Tb*GH2), 10 μL of diluted enzyme solution were added. The hydrolysis was stopped after 10 min by adding 100 μL of 100 mM Na_2_CO_3_. The absorbance was measured at 410 nm. All measurements were determined in triplicates. About 1 U of enzyme activity was defined as the amount of enzyme needed for the release of 1 μmol 4-nitrophenol per minute. Kinetic parameters were determined according to Michaelis and Menten (1913).

### Effect of pH and temperature

Relative activities against 4-Np-β-d-glucopyranoside (2 mM) were measured in the range of pH 4.0–10.0 using 20 mM Britton–Robinson buffer (Britton and Robinson, 1931). To determine the pH stability, both enzymes were preincubated in 20 mM Britton–Robinson buffer, pH 3.0–10.0 at 4°C for 24 h. To stabilize the enzymes, the concentration of the solution was adjusted to 0.1 mg/mL with BSA. After preincubation, the relative activity toward 4-Np-β-d-glucopyranoside (2 mM) was determined in 20 mM Britton–Robinson buffer pH 6.5 and 95°C (*Ta*GH2), or in 20 mM maleate buffer at 90°C (*Tb*GH2).

The influence of temperature was determined by measuring relative enzyme activities in the range of 10–115°C with 4-Np-β-d-glucopyranoside (2 mM) in 20 mM citrate buffer (*Ta*GH2) or 20 mM maleate buffer (*Tb*GH2), pH 6.5. To examine the temperature stability, both enzymes were preincubated at 50–90°C for 0–24 h in 20 mM citrate buffer (*Ta*GH2) or 20 mM maleate buffer (*Tb*GH2), pH 6.5. The protein concentration of the solution was adjusted to 0.1 mg/mL with BSA. After preincubation, the relative activity toward 4-Np-β-d-glucopyranoside (2 mM) was determined in 20 mM citrate buffer (*Ta*GH2) or 20 mM maleate buffer (*Tb*GH2), pH 6.5, at 95°C (*Ta*GH2) and 90°C (*Tb*GH2).

### Determination of hydrolysis products

Hydrolysis products of 1% (w/v) cellobiose and lactose were examined by HPLC (Agilent Technology 1260 Infinity Quarternary LC system with 1260 ALS sampler, 1260 Quat pump and 1260 R_i_ detector). The enzymes were incubated with the substrates at 90°C for 1 h in 20 mM citrate buffer (*Ta*GH2) or 20 mM maleate buffer (*Tb*GH2), pH 6.5. After hydrolysis, 20 μL of the centrifuged and filtered solution was applied to a Hi-Plex H column (Agilent Technologies, Waldbronn, Germany). Water was used as solvent with a flowrate of 0.6 mL/min. To identify the hydrolysis products, the retention times (min) were compared to the standards cellobiose (9.433), lactose (9.789), and glucose (11.247).

### Nucleotide sequence accession number

DNA sequences of *tagh2* and *tbgh2* were deposited in GenBank (*tagh2*: HG969993, *tbgh2*: HG969994).

## Results

### Identification of novel GH-encoding genes

Gene libraries were constructed from pure cultures of *T. antranikianii* and *T. brockianus*, and screened for genes coding for enzymes active toward esculin. One activity-conferring *E. coli* clone was detected from each library. The respective inserts of the phagemids (pBK-CMV:*Ta* and pBK-CMV:*Tb*) harbored *tagh2* and *tbgh2*. The nucleotide sequences of *tagh2* and *tbgh2* were 80% identical. GC contents of 68.4 and 66.7% with GC proportions in the third codon position (GC_3_) of 86.3 and 82.3% were observed.

The deduced amino acid sequences *Ta*GH2 and *Tb*GH2 possessed theoretical isoelectric points (pI) of 6.1 (*Ta*GH2) and 5.99 (*Tb*GH2). Molecular masses of 79.0 kDa (*Ta*GH2) and 78.1 kDa (*Tb*GH2) were computed. The proteins were compared to proteins annotated in databases (GenBank). *Ta*GH2 exhibited highest identity (98%) to a putative β-galactosidase from *Thermus scotoductus* (YP_004203085, Table 1, Row 3). *Tb*GH2 showed 82% identity to a hypothetical protein from *Thermus* sp. (YP_005653839, Table 1, Row 4). The annotated proteins were deduced from ORFs identified in the course of whole genome sequencing projects of different *Thermus* species. Additionally, identities of 86% (*Ta*GH2) and 83% (*Tb*GH2) were obtained by comparison with a putative GH2 β-mannosidase from *Thermus thermophilus* (ACH89346, Table 1, Row 6). Identities to annotated proteins from *Thermus* sp. ranged from 83 to 98% (*Ta*GH2) and from 80 to 83% (*Tb*GH2). GH2 enzymes from genera other than *Thermus* were observed with identities of 38–42% (*Caldilinea aerophila*, YP_005442964 and *Alicyclobacillus pohlia*, WP_018133520.1; Table 1, Rows 7 and 8).

**Table 1:** **Identities of *Ta*GH2 and *Tb*GH2 to other GH2-proteins**.

	1.	2.	3.	4.	5.	6.	7.	8.	9.
1. *T. antranikianii Ta*GH2	100%								
2. *T. brockianus Tb*GH2	80% (100)	100%							
3. *T. scotoductus* β-galactosidase (YP_004203085.1)	98% (100)	80% (100)	100%						
4. *Thermus* sp. hyp. Protein (YP_005653839.1)	83% (100)	colorno882% (100)	83% (100)	100%					
5. *T. igniterrae* β-galactosidase (WP_018110934.1)	82% (100)	81% (100)	81% (100)	83% (100)	100%				
6. *T. thermophilus* put. β-mannosidase (ACH89346.1)	86% (92)	83% (93)	85% (99)	84% (99)	86% (87)	100%			
7. *A. pohliae* put. Protein (WP_018133520.1)	38% (97)	41% (83)	38% (90)	38% (95)	38% (94)	41% (92)	100%		
8. *C. aerophila* put. GH (YP_005442964.1)	41% (76)	42% (82)	41% (78)	41% (84)	42% (83)	41% (96)	45% (99)	100%	
9. *E. coli* LacZ (1HN1_D)	27% (35)	29% (32)	27% (37)	27% (32)	29% (46)	28% (46)	23% (61)	23% (58)	100%

*Enzymes from *Thermus* spp. are highlighted in gray. Query-coverage values are indicated in % in brackets*.

*Numbers at the top are listed in the left column*.

**Figure 1:**
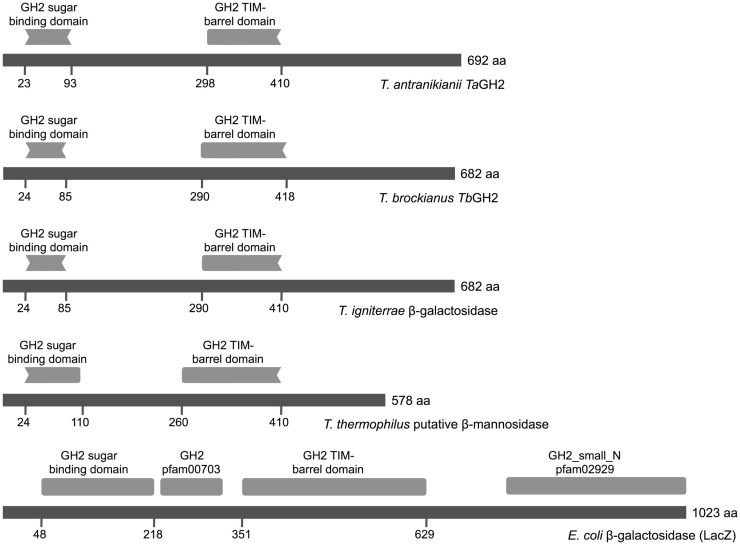
**Conserved domains of GH2 family enzymes**. The amino acid sequences of *Ta*GH2 (*T. antranikianii*), *Tb*GH2 (*T. brockianus*), β-galactosidase (*T. igniterrae*, WP_018110934.1), putative β-mannosidase (*T. thermophilus*, ACH89346), and LacZ (*E. coli*, 1HN1_D) are depicted in dark gray with homologous predicted sugar-binding domain (pfam02837) and TIM-barrel domain (pfam02836, Conserved Domain Database) in light gray.

**Figure 2:**
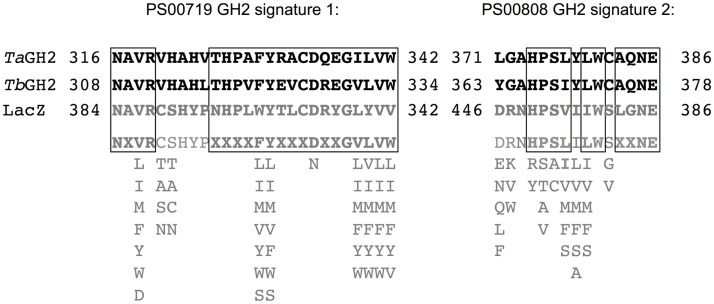
**Amino acid signatures of GH2 family enzymes**. The amino acid sequences of *Ta*GH2 and *Tb*GH2 are shown in black and the sequence of LacZ (*E. coli*, 1HN1_D) and the consensus pattern of the two characteristic signatures of GH2 are depicted in gray with possible residue substitutions indicated below (http://prosite.expasy.org/PDOC00531). Corresponding amino acids are shown in bold characters and conformity within all sequences is framed.

Notably, the Conserved Domain Database indicated two incomplete GH2 domains for both enzymes (Figure 1). A partial GH2 sugar-binding domain (pfam02837) in the protein sequence of *Ta*GH2 (aa 23–93) and *Tb*GH2 (aa 24–85), and an incomplete GH2 TIM-barrel domain (pfam02836) within *Ta*GH2 (aa 298–410) and *Tb*GH2 (aa 290–418) were predicted. Comparable domains were described for *E. coli* β-galactosidase LacZ in a complete form with low identities to the TIM-barrel domain (≤ 30%) and to the sugar-binding domain (≤ 20%) of *Ta*GH2 and *Tb*GH2 (Figure 1). Within LacZ, the consensus patterns N-X-[LIVMFYWD]-R-[STACN](2)-H-Y-P-X(4)-[LIVMFYWS](2)-x(3)-[DN]-X(2)-G-[LIVMFYW](4) and [DENQLF]-[KRVW]-N-[HRY]-[STAPV]-[SAC]-[LIVMFS]-[LIVMFSA]-[LIVMFS]-W-[GSV]-X(2,3)-N-E, which were described as conserved signatures of GH2, were identified^6^ (Figure 2). Principally, these conserved signatures were also detected as variations within *Ta*GH2 and *Tb*GH2.

### Expression of *tagh2* and *tbgh2* and protein purification

**Figure 3:**
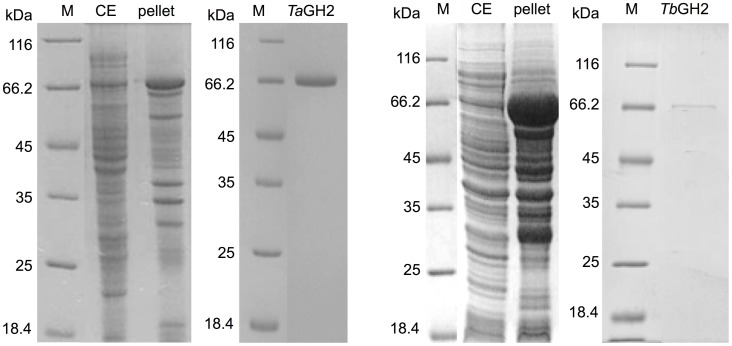
**SDS-PAGE analysis of crude extracts and purified *Ta*GH2 and *Tb*GH2**. Soluble (CE) and insoluble (pellet) fractions of crude extracts and purified proteins were separated by SDS-PAGE (200 V, 50 min, M: Marker).

The genes *tagh2* and *tbgh2* were amplified and expressed in *E. coli* BL21 Star^TM^(DE3) using pQE-80L (Figure 3). A high proportion of *Tb*GH2 was produced in insoluble form. *Ta*GH2 and *Tb*GH2 were purified from soluble crude extracts. After heat precipitation and Ni-NTA affinity chromatography, *Ta*GH2 was homogenous with a yield of 17.3%, whereas *Tb*GH2 was subsequently subjected to size exclusion chromatography with a yield of 6.7%. The calculated molecular masses of 79.0 kDa (*Ta*GH2) and 78.1 kDa (*Tb*GH2) were confirmed by SDS gel analysis (Figure 3).

### Substrate conversion and kinetics of *Ta*GH2 and *Tb*GH2

**Figure 4:**
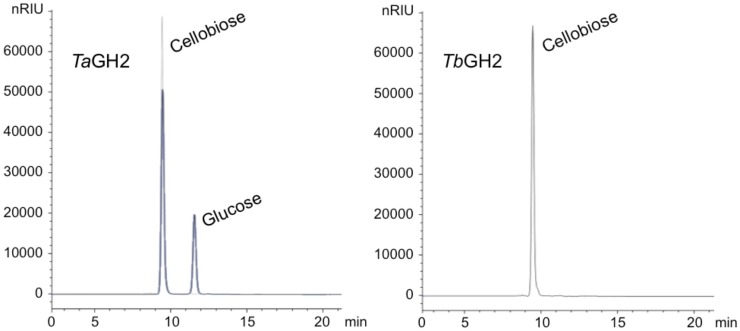
**HPLC analyses of cellobiose degradation**. The degradation of cellobiose (1% w/v) by *Ta*GH2 and by *Tb*GH2 is depicted. The hydrolysis was carried out at 90°C for 1 h in 20 mM citrate or maleate buffer (pH 6.5). Subsequently, the samples were subjected to HPLC analyses using an Hi-Plex H column and water with a flow rate of 0.6 mL/min. Standards are shown in gray. nRIU, nano Refractive Index Units.

The purified enzymes *Ta*GH2 and *Tb*GH2 were analyzed regarding their substrate specificity. The predicted domains of GH2 and the highest homologies to annotated β-galactosidases suggested both enzymes being β-galactosidases. Nitrophenol-linked substrates were tested and in contrast to expectations, *Ta*GH2 and *Tb*GH2 exhibited highest activities toward 4-Np-β-d-glucopyranoside (*Ta*GH2: 3,966 U/mg and *Tb*GH2: 660 U/mg), and residual activities of 40 and 51% against 4-Np-β-d-galactopyranoside. In accordance, Michaelis constants (*K*_m_) were lower when 4-Np-β-d-glucopyranoside was used as substrate (Table 2). The catalytic efficiency was 29,532 mM^−1^ s^−1^ for *Ta*GH2 and 7,797 mM^−1^ s^−1^ for *Tb*GH2. The maximum reaction rate (*V*_max_) of *Ta*GH2 was higher when measured with 4-Np-β-d-glucopyranoside (4,028 ± 21 μmol min^−1^ mg^−1^) compared to 4-Np-β-d-galactopyranoside (2,615 ± 3 μmol min^−1^ mg^−1^). On the contrary, *Tb*GH2 exhibited a higher maximum reaction rate toward 4-Np-β-d-galactopyranoside (1,333 ± 11 μmol min^−1^ mg^−1^) than toward 4-Np-β-d-glucopyranoside (778 ± 22 μmol min^−1^ mg^−1^). Other nitrophenol-linked substrates were not converted. Furthermore, non-nitrophenolic substrates, such as cellobiose and lactose (1%, w/v), were tested. Interestingly, *Ta*GH2 converted cellobiose (Figure 4). *Tb*GH2 acted neither on cellobiose nor on lactose.

**Table 2:** **Kinetic parameters of *Ta*GH2 and *Tb*GH**.

	4-Np-β- glucopyranoside		4-Np-β- galactopyranoside	
	*Ta*GH2	*Tb*GH2	*Ta*GH2	*Tb*GH2
*K*_m_ [mM]	0.73 ± 0.03	0.50 ± 0.02	2.20 ± 0.02	3.56 ± 0.07
*V*_max_ [μmol min^−1^ mg^−1^]	4,028 ± 21	778 ± 22	2,615 ± 3	1,333 ± 11
*k*_cat_ [s^−1^]	21,559	3,899	13,996	6,683
*k*_cat_/*K*_m_ [mM^−1^ s^−1^]	29,532	7,797	6,361	1,877

*Activity of *Ta*GH2 and *Tb*GH2 was measured at 90°C in 20 mM citrate or maleate buffer (pH 6.5) with 0–10 mM 4-Np-β-D-glucopyranoside and 0–25 mM 4-Np-β-D-galactopyranoside for 10 min*.

### Influence of temperature and pH

**Figure 5:**
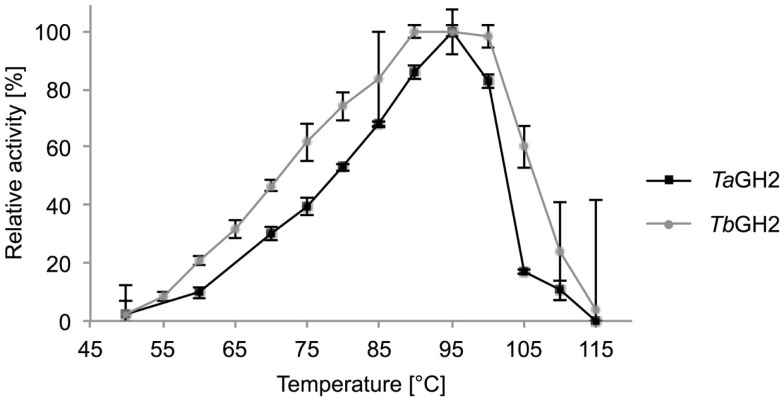
**Effect of temperature on activity of *Ta*GH2 and *Tb*GH2**. Activity of *Ta*GH2 and *Tb*GH2 was measured at different temperatures (50–115°C) for 10 min in 20 mM citrate or maleate buffer (pH 6.5) with 2 mM 4-Np-β-D-glucopyranoside. Temperatures above 95°C were measured in heated oil.

**Figure 6:**
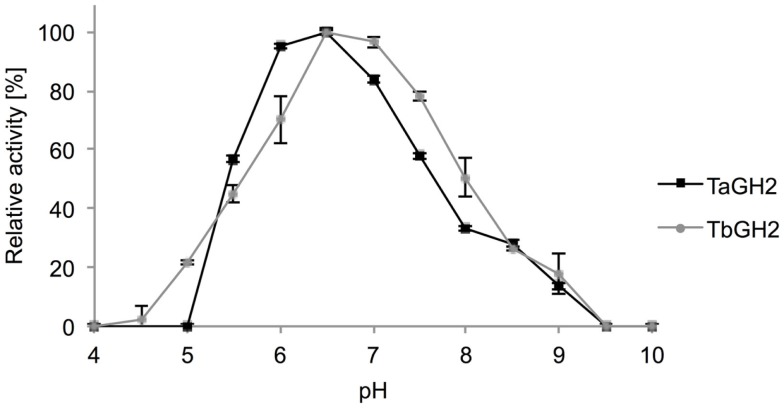
**Effect of pH on activity of *Ta*GH2 and *Tb*GH2**. Activity of *Ta*GH2 and *Tb*GH2 was measured for 10 min at 95 or 90°C in 20 mM Britton–Robinson buffer (pH 4–10) with 2 mM 4-Np-β-D-glucopyranoside.

Highest activities toward 4-Np-β-d-glucopyranoside were observed at 95°C (*Ta*GH2) and 90°C (*Tb*GH2) at pH 6.5 (Figures 5 and 6). Residual activities of 83 ± 2% (*Ta*GH2) and 98 ± 4% (*Tb*GH2) were detected at 100°C. *Ta*GH2 showed no loss of activity when incubated at 50–90°C for 3 h (Table 3). By contrast, *Tb*GH2 was less stable and showed 17 ± 6% activity at 80°C after 3 h of incubation. At 70°C, *Ta*GH2 exhibited a half-life time of approximately 22 h and *Tb*GH2 of 12 h. In accordance, the predicted *T*_m_ was higher for *Ta*GH2 (>65°C) when compared to *Tb*GH2 (55–65°C) (Ku et al., 2009). Thermal activation was observed for both enzymes at 50 and 60°C. The enzyme *Ta*GH2 was stable when preincubated (4°C, 24 h) at pH values between 3.0 and 10.0 (81 ± 5-100 ± 2%). *Tb*GH2 was stable at pH 3.5–7.5 (71 ± 8-114 ± 13%) with residual activities of 63 ± 7 and 52 ± 11% at higher pH values (pH 8.5, 9.5).

**Table 3:** **Effect of temperature on stability of *Ta*GH2 and *Tb*GH2**.

	3 h		24 h	
	*Ta*GH2	*Tb*GH2	*Ta*GH2	*Tb*GH2
50°C	127 ± 5	129 ± 9	151 ± 4	246 ± 12
60°C	123 ± 2	199 ± 7	108 ± 8	202 ± 10
70°C	123 ± 4	94 ± 3	47 ± 3	0
80°C	128 ± 2	17 ± 6	15 ± 3	0
90°C	127 ± 3	0	0	0

*Activity of *Ta*GH2 and *Tb*GH2 was measured at 90°C in 20 mM citrate or maleate buffer (pH 6.5) with 2 mM 4-Np-β-D-glucopyranoside for 10 min after preincubation for 3 or 24 h, respectively, at different temperatures (50–90°C). Activities determined at 0 h were set to 100%*.

### Influence of additives

The influence of different metal ions on the activity of *Ta*GH2 and *Tb*GH2 was tested (Table 4). K^+^, Mg^2+^, and Na^+^ ions (1–10 mM) had no negative influence on the enzymatic performance of both enzymes. Furthermore, Ca^2+^, Co^2+^, Mn^2+^, Ni^2+^, Rb^+^, and Sr^2+^ (1–10 mM) had no significant influence on the hydrolytic action of *Ta*GH2 (88 ± 4-104 ± 4%). The activity of *Tb*GH2 was reduced in presence of 10 mM CaCl_2_ (56 ± 20%), MnCl_2_ (64 ± 12%), RbCl (81 ± 5%), and SrCl_2_ (67 ± 15%), or completely inhibited in presence of 5–10 mM CoCl_2_ and NiCl_2_. *Tb*GH2 was inactivated by 1 mM Ag^+^, Cu^2+^, and Zn^2+^. *Ta*GH2 was exclusively inhibited by Ag^+^. Fe^2+^ and Fe^3+^ (1 mM) had no influence on the enzymatic performance of both hydrolases (95 ± 2-120 ± 2%). *Tb*GH2 showed higher activity when 1 mM AlCl_3_ (149 ±1%), CrCl_3_ (139 ± 4%), KCl (146 ± 4%), MgCl_2_ (136 ± 4%), NaCl (138 ± 11%), or RbCl (143 ± 10%) was added.

**Table 4:** **Effects of metal ions on the activity of *Ta*GH2 and *Tb*GH2**.

Reagent	Relative activity of *Ta*GH2 (%)			Relative activity of *Tb*GH2 (%)		
	1 mM	5 mM	10 mM	1 mM	5 mM	10 mM
None	100 ± 3	100 ± 5	100 ± 2	100 ± 4	100 ± 4	100 ± 5
AgNO_3_	0	0	0	0	0	0
AlCl_3_	97 ± 4	53 ± 1	17 ± 5	149 ± 1	98 ± 13	0
CaCl_2_	100 ± 3	100 ± 4	103 ± 4	93 ± 6	72 ± 5	56 ± 20
CoCl_2_	99 ± 3	101 ± 2	97 ± 2	52 ± 7	0	0
CrCl_3_	88 ± 2	43 ± 3	13 ± 5	139 ± 4	53 ± 11	0
CuCl_2_	98 ± 4	64 ± 5	20 ± 4	0	0	0
FeCl_2_	97 ± 1	42 ± 3	0	117 ± 3	31 ± 14	0
FeCl_3_	95 ± 2	53 ± 4	12 ± 3	120 ± 2	37 ± 18	0
KCl	99 ± 3	98 ± 3	102 ± 4	146 ± 4	144 ± 7	83 ± 5
MgCl_2_	99 ± 1	103 ± 2	103 ± 2	136 ± 4	117 ± 2	113 ± 7
MnCl_2_	96 ± 3	98 ± 1	88 ± 4	94 ± 15	86 ± 3	64 ± 12
NaCl	97 ± 2	99 ± 4	100 ± 4	138 ± 11	129 ± 6	104 ± 10
NiCl_2_	99 ± 3	104 ± 4	90 ± 3	61 ± 13	0	0
RbCl	96 ± 2	102 ± 1	98 ± 1	143 ± 10	143 ± 2	81 ± 5
SrCl_2_	101 ± 3	104 ± 4	104 ± 2	127 ± 4	106 ± 9	67 ± 15
ZnCl_2_	99 ± 2	97 ± 2	64 ± 3	0	0	0

*Activity of *Ta*GH2 and *Tb*GH2 was measured at 90°C in 20 mM citrate or maleate buffer (pH 6.5) with 2 mM 4-Np-β-D-glucopyranoside for 10 min in presence or absence of metal ions*.

The presence of 5 mM additives, such as β-mercaptoethanol, DTT, EDTA, urea, iodoacetic acid, sodium azide, and Tween 80 did not decrease the activity of both enzymes. Moreover, Triton X-100 and Tween 20 did not influence the performance of *Tb*GH2, whereas *Ta*GH2 exhibited 49 ± 2 and 76 ± 1% residual activity. Complete loss of activity for both glycoside hydrolases was detected when SDS or CTAB (5 mM) were added.

## Discussion

Screening of gene libraries from *T. antranikianii* and *T. brockianus* on esculin resulted in the identification of two novel glycoside hydrolases (*Ta*GH2 and *Tb*GH2). Identities of amino acid sequences higher than 80% were observed to proteins from other *Thermus* species. The glycoside hydrolase family 2 domains (sugar binding domain and TIM-barrel domain) appeared to be incomplete. The domains are either truncated but functional or they are not recognized as domains due to large differences to other representatives of GH2. Comparison with the GH2 LacZ from *E. coli* (1HN1_D) showed low similarities within the corresponding domains. Additionally, conserved amino acid signatures typical for members of GH2 were partially identified (Figure 2). Similar hypothetical proteins with typical conserved domains from different genera were detected with identities of 39% (*Ta*GH2) and 42% (*Tb*GH2) or below. Thus, a differentiation of proteins produced from *Thermus* spp. would be suggested. Low sequence similarities within one protein superfamily could be the result of adaptation to different environmental conditions. Different enzymatic activities may have developed from a common ancestor (Galperin and Koonin, 2012). Structural diversification that preserved the active site residues may result in catalytically active enzymes with altered substrate specificity. Evolutionary pressure promotes functional and effective enzymes, which may result in reduction of conserved domains with remaining functionality (Juers et al., 1999). However, substrate specificity can be neglected for classification of glycoside hydrolases, since the structural homologies especially the motif forming the catalytic center is highly conserved (Henrissat and Davies, 1997). Homologies of up to 98 and 82%, respectively, were observed by comparison with annotated β-galactosidases or β-mannosidases from *Thermus* species. No similar characterized enzyme to *Ta*GH2 or *Tb*GH2 was detected in the NCBI database; thus, classification of similar proteins was achieved by domain prediction rather than by functional analyses. Highest catalytic efficiencies for *Ta*GH2 and *Tb*GH2 were observed toward 4-Np-β-d-glucopyranoside with residual activities toward 4-Np-β-d-galactopyranoside. Cellobiose conversion distinguishes *Ta*GH2 from *Tb*GH2. The hydrolysis characteristics may vary between artificial and natural substrates. This may be due to differences in size, charge, and binding properties of the artificial compound. Higher activity toward artificial substrates was also described as common phenomenon for α-galactosidases (Wang et al., 2014). Hydrolysis of cellobiose or lactose by *Tb*GH2 could not be detected, although high β-glucosidase activity was observed when the artificial substrate was used. β-Glucosidases are reported to often exhibit a broad substrate spectrum. A β-glucosidase from *Thermotoga neapolitana* hydrolyzed among others 4-Np-β-d-glucopyranoside, cellobiose, and lactose (Park et al., 2005). Likewise, a GH1-β-glucosidase derived from a hot-spring metagenome exhibited activity toward 4-Np-β-d-glucopyranoside, 4-Np-β-d-galactopyranoside, cellobiose, and lactose (Schröder et al., 2014). However, the newly discovered GH2 β-glucosidases *Ta*GH2 and *Tb*GH2 showed a narrow substrate range.

Protein production in the mesophilic host *E. coli* resulted in notable amount of inclusion bodies due to differences in genomic GC contents of the genus *Thermus*, as previously reported (Ishida and Oshima, 1994; Fridjonsson et al., 1999). The β-glucosidase *Ta*GH2 and the β-glucosidase/galactosidase *Tb*GH2 showed high activities at elevated temperatures. Other β-glycosidases from *Thermus thermophilus* also show activity at 88–90°C and pH 5.4–7.0 (Dion et al., 1999; Nam et al., 2010). A thermal activation was detected at 50 and 60°C, especially for *Tb*GH2 with 246 and 202% activity after 24 h, respectively. It was reported in the literature that enzymes from thermophilic organisms produced in mesophilic hosts may require thermal activation as demonstrated for a β-glycosidase from *T. thermophilus* at 70°C (Gerard et al., 2002). Specific activities were considered high with 3,966 and 660 U/mg, when compared to the majority of previously reported β-glucosidases and β-galactosidases with <1–100 U/mg^7^. Similar to GH family 1 β-glycosidases from *T. thermophilus*, significantly lower *K*_m_ values were observed with 4-Np-β-glucopyranoside when compared to -galactopyranoside (Dion et al., 1999; Nam et al., 2010). Highest substrate affinities for -glucopyranoside were also reported for other β-glucosidases from an uncultured soil bacterium and *Cellulomonas flavigena* with *K*_m_-values of 0.16 and 7.1 mM, respectively (Barrera-Islas et al., 2007; Kim et al., 2007). Hence, the enzymes described here exhibit *K*_m_-values in the range of other described GH1 β-glucosidases.

Ag^+^, Cu^2+^, and Fe^3+^ are most frequently described as glycoside hydrolase inhibitor (Cairns and Esen, 2010). By contrast, Fe^3+^ (1 mM) had no influence on the activity of both enzymes. Zinc ions inhibited *Tb*GH2 completely but had, interestingly, no effect on activity of *Ta*GH2. The surfactant CTAB (5 mM) inhibited both enzymes. The negatively charged catalytic amino acids in the active center might be covered by the positively charged additive. Moreover, negatively charged surface residues might be influenced resulting in structural destabilization of the enzyme. Although three cysteins are present in the amino acid sequence of both enzymes, reducing agents such as DTT and β-mercaptoethanol did not show a negative effect on the activity. Either no disulfide bond is formed or it is not affected by the prevalent conditions. It is a frequently described phenomenon that reducing agents can also have stabilizing effects on enzymes (Park et al., 2005). The influence of additives on β-glucosidases and β-galactosidases does not follow a certain pattern, and appears to be specific for each individual enzyme.

*Ta*GH2 and *Tb*GH2 were classified as members of glycoside hydrolase family 2 based on amino acid sequence similarities. Although GH family 2 comprises enzymes with different substrate specificities, β-glucosidase activity has not been reported in this family, yet (Henrissat, 1991). This finding proves the necessity of function-based screening to identify genes coding for proteins with unusual domain structures or unexpected activities. The substrate specificity was reported to be less relevant than structural homologies for classification of members of glycoside hydrolase families (Henrissat and Davies, 1997).

In conclusion, our study demonstrates that enzymes structurally related to GH family 2 can exhibit more diverse substrate specificities than previously predicted. Therefore, we recommend to incorporate β-glucosidases into GH family 2 and consequently to evaluate activity on glucopyranosides including cellobiose for characterization of enzymes from this family.

## Conflict of Interest Statement

The authors declare that the research was conducted in the absence of any commercial or financial relationships that could be construed as a potential conflict of interest.

## Footnotes

^1^http://www.cazy.org/

^2^http://nocardia.nih.go.jp/fp4/

^3^http://www.ncbi.nlm.nih.gov/

^4^http://www.ebi.ac.uk/Tools/pfa/iprscan/

^5^http://www.ebi.ac.uk/Tools/msa/clustalw2/

^6^http://prosite.expasy.org/PDOC00531

^7^http://www.brenda-enzymes.org
